# Assessment of Smoking Behaviour of Adolescents in Two Districts of Nepal and Implications of the MPOWER Policy Measures: A Mixed Method Study

**DOI:** 10.31729/jnma.8575

**Published:** 2024-05-31

**Authors:** Jemish Acharya, Chardsumon Prutipinyo, Nithat Sirichotiratana, Kwanjai Amnatsatsue

**Affiliations:** 1 Department of Global Health, Faculty of Public Health, Mahidol University, Bangkok, Thailand; 2 Department of Public Health Administration, Faculty of Public Health, Mahidol University, Bangkok, Thailand; 3 Department of Public Health Nursing, Faculty of Public Health, Mahidol University, Bangkok, Thailand

**Keywords:** *global youth tobacco survey*, *MPOWER Policy*, *tobacco control*

## Abstract

**Introduction:**

Tobacco use is the single most preventable cause of death and disease worldwide. This study aimed to assess the smoking behaviour of adolescents in two districts of Nepal and examine the implications of the MPOWER policy on their smoking behaviour.

**Methods:**

An explanatory, mixed-method study was conducted in two districts of Nepal. Qualitative in-depth interviews were conducted among nine participants aged 35-50, representatives of the Ministry of Health, academic institutions, and managers of organisations working in tobacco control, using snowball sampling method. A total of 306 students of age 13-15 years, from six government schools were recruited through simple random sampling method using an adapted version of the Global Youth Tobacco Survey (GYTS). Findings of the qualitative and quantitative study were explained for concurrence and relevance to present overall study findings. Ethical approval was received from the Nepal Health Research Council and Mahidol University.

**Results:**

Findings from the survey reported that a total of 25 (8.10%) of respondents were smokers, from which 13 (4.20%) were current smokers and 12 (3.90%) were ever smokers. Socio-economic status played a crucial role in the smoking behaviour. Although the survey among adolescents indicated an awareness of the policies, there was a lack of cessation services, which was concurrent with the qualitative findings. The interviews recommended improvements in the implementation of policy ban on public smoking, taxation, and availability of cessation services.

**Conclusions:**

The MPOWER policies are not regulated strictly, especially in areas of the ban on public smoking, regulating the selling of cigarettes to adolescents <18 years, and availability of cessation services.

## INTRODUCTION

More than 6 million people per year die from tobacco use across the globe.^12^ One-third of the world's population, aged 15 years and older, are smokers.^3,4^ The Wolrd Health Organization Framework Convention on Tobacco Control (WHO-FCTC) provides the foundation for countries to implement and manage tobacco control.^5^ To help make this a reality, WHO introduced the MPOWER measures set up to facilitate delivery of the (WHO-FCTC), country-wide. This framework also addresses the social, economic, and environmental impact of tobacco. The Government of Nepal assented to the directives of the Tobacco Product Control and Regulatory Act 2010 in November 2011.^6,7^

Despite the alarming increase in the proportion of smokers in the younger population, there are few documentations and consistent lack of a periodic survey. There is insufficient research conducted to draw evidence on the implications of MPOWER policies.

The aim of this study was to assess the smoking behaviour of adolescents and examine the implications of the MPOWER policy on their smoking behaviour.

## METHODS

An explanatory, mixed-method study design using a simple random sampling method was used. The study was conducted between December 2020 to April 2021 in Jhapa and Kathmandu, Nepal. Prior consent was obtained from the interviewees and the students. The participants privacy was maintained during the interviews. The procedure of the study was submitted to be reviewed and approved by the ethics committee of Mahidol University, Bangkok, and ethical approval was also obtained from Nepal Health Research Council (Reference number: 33).

Qualitative study: Nine in-depth interviews were conducted using a semi-structured guide, to explore participants' perceptions of the implementation and enforcement of the MPOWER policy, and their views on how the policy has affected smoking status among the students. Interviewees comprised officials age 35-50 years, from the Ministry of Health and Population academic researchers, non government organization officials, with representation from Jhapa and Kathmandu districts of Nepal. They were identified through snowball sampling and those fitting the criteria were included after we obtained consent from them. Respondents were individuals who were involved during the Tobacco Control Act implementation in 2010 and are currently involved in tobacco control. Interview guideline was constructed based on literature, the current situation of tobacco control policies, and the smoking situation in Nepal post-implementation of Tobacco control. Guidelines were constructed based on the MPOWER Package of Tobacco Control provided by WHO-FCTC for party countries to implement tobacco control measures under the following major domains:^5^

i. Monitor tobacco use and prevention policies
ii. Protect people from second hand smoke,
iii. Offer help to quit tobacco,
iv. Warn about ill effects of tobacco,
v. Enforce bans on tobacco advertising and sponsorship,
vi. Reinforce taxes on tobacco.

In-depth interview guidelines were used for the interviews. Interviews were recorded manually and notes were taken and a recorder was used after obtaining consent where applicable for the purpose of transcription and translation into English later on.

Transcripts were labelled, sorted, synthesized, and analyzed using key thematic words. Anonymity and confidentiality of information was maintained. The findings/themes were validated through triangulations of data across sources, considering the characteristics of the informants incorporating a wide range of different perspectives. The guideline was verified by public health experts, researchers, and tobacco control policy experts working in the local area. Wherever possible, triangulation was used to assure the quality of the study.

Document analysis matrix: Using the WHO-FCTC MPOWER measures guidelines, all tobacco control measures were identified The matrix was formed by rows of the 6 themes corresponding to the MPOWER Policies in each category, which was applied for presenting the results.^5^

i. Monitor tobacco use and prevention policies
ii. Protect people from second hand smoke,
iii. Offer help to quit tobacco,
iv. Warn about ill effects of tobacco,
v. Enforce bans on tobacco advertising and sponsorship,
vi. Reinforce taxes on tobacco.

Document analysis process: Using the document analysis matrix as per WHO-FCTC MPOWER guidelines, relevant information was gathered through the interviews. Compilation of the transcripts based on the 6 measures was identified from the matrix.

Data Triangulation: Upon completion of qualitative, compilation and reading of the transcripts was done for data familiarization. General codes were identified from the transcripts and categorized into 6 major areas. Main themes and sub-themes once emerged from the interviews were identified and effort was made to triangulate the information from the interviews and literature reviews. The thematic keywords at the time of the interview were highlighted and rearranged as per the 6 major policies provided by WHO-FCTC MPOWER, after which the information under particular headings was reviewed and interpreted. Content analysis was done. NVivo Pro 12 was used for the analysis of qualitative data.

Quantitative study: A self-administered questionnaire survey among 306 secondary school students was then conducted, using the Global Youth Tobacco Survey Questionnaire and results were presented with a focus on current smoking students. The outcome variables were current smokers and non-smokers. A translated, validated version of the Global Youth Tobacco Survey Questionnaire (GYTS) was used for the survey.^1^ Students for the quantitative survey were selected from two geographical regions of Nepal, using simple random sampling method. For the selection of districts, firstly, two regions (Terai and Kathmandu) were chosen in random. From among the regions, one district from each region was chosen by lottery method. Following that, three secondary schools were selected from each district. GYTS sampling frames have excluded schools with enrollments below 40 students. Only schools that contain grades associated with secondary school students of age 13-15 years were selected. Grades were selected systematically to include one grade from each school and 3 schools from each district. A total of 6 schools were included from 2 districts. We excluded those who were unwilling to participate and did not provide consent. Being a school-based survey, consent was obtained from the school authorities after providing an information sheet for circulation to parents. For the quantitative survey, students aged 13-15 years studying in government schools of selected districts and those who consented to participation were included in the survey.

**Sample size for quantitative study:**

The sample size estimation was conducted using the following formula


$$\begin{array}{ll} \text{n} & ={\text{Z}}^{2}\times \frac{\text{p}\times \text{q}}{{\text{e}}^{\text{2}}}\\ & =1.96^{2}\times \frac{0.23\times 0.77}{0.05^{2}}\\ & =272 \end{array}$$

Where,

n= minimum required sample size
Z= 1.96 at 95% Confidence Interval (CI)
p= prevalence taken as 23% from the previous study^8^
q= 1-p
e= margin of error, 5%

Using the formula for sample size estimation by proportion, the minimum required sample size is 272. A 10% excess was included to cover withdrawal issues which gives us a total of a minimum of 300 students to be recruited in the study. A minimum of 50 students from each grade in each district were recruited for the study. In the end, 306 students were recruited. Descriptive statistics such as mean, percentage, and standard deviation were calculated. All analysis was conducted in Statistical Package for Social Science Version 20.

## RESULTS

Qualitative findings: Among our participants in the interviews, six of them had 5-10 years of experience in the current position, which was in tobacco control, two of them had more than 10 years of working experience in tobacco control, and one had less than 5 years in the role of a tobacco control manager. The key findings of thematic analyses are presented below :

**a. Cultural acceptance of smoking among students**

Smoking is acceptable in Nepalese culture, particularly as social recognition. Smokeless tobacco, beedi (selfcultivated and rolled tobacco) have been a part of the Nepalese culture for centuries, used in celebrations and festivals.

"Cigarettes are modern but bidi was already there. That is why it is a question why it is high there" (Academic researcher, Jhapa)

Smoking status has been perceived to be generally low in Nepal in youths and student groups, compared to adults. Initiation of smoking usually happens during 10-15 years of age and the new smokers are usually students, in both urban and rural areas.

"Students learn to smoke in schools mostly, through their friends and teachers going to events and parties and while coming and going to school, and mostly in teenage." (Policy maker, Kathmandu)

**b. Easy Access to smoking**

One finding that emerged was about easy access to cigarettes. In Nepal, where there is a farming predominance, people can farm tobacco, make their own cigarettes, and smoke. This was mostly seen in the rural areas and especially this was practiced in other hand-rolled forms of smoking and smokeless forms of tobacco like Beedi, and Khaini.

"When we look at the rural, mostly, even if there is no accessibility, there is homemade bidi."(NGO Manager, Kathmandu)

**C. Social determinants of smoking**

**Setting or areas of residence:** The setting or where the people live was seen to be an important finding which plays a role in the smoking situation in Nepal. Urban areas like Kathmandu show a higher prevalence of cigarette smokers compared to the rural areas. Factors like in-country migration from all over the country into Nepal and then subsequently into urban areas for jobs and education were reported.

"Rural population that shifts into the urban population, when that happens, they carry the risk factor with themselves. It is one of the reasons why it seems higher in the urban population" (Academic researcher, Kathmandu)

**Gender:** The smoking situation among females was seen to be relatively low compared to that of males, even in the urban areas, consistent with qualitative findings.

"Females are seen less smoking on the streets than males. Very less, isn't it? But maybe they are hiding and smoking because it is still taboo to see girls smoking. If you ask them alone, in confidence they will say yes, maybe." (Academic researcher, Jhapa)

**Socioeconomic status:** Similarly, lower socio-economic status presents with a higher proportion of smoking. Some reasons for this were cited as a possible lack of awareness about the ill effects of smoking and knowledge regarding harm and sometimes smoking was seen to be a result of the stress that came with being socio-economically deprived. However, in the urban areas, sometimes, people from higher socioeconomic status smoked more as a trend or a trickle-down effect of modernization.

"When there is higher socio economic, there is higher education and mostly education is higher among higher socio-economic. Maybe that's why there is lower consumption among higher socio economic and vice versa. Another reason could be stress"(NGO manager, Kathmandu)

**Implication of the MPOWER policy measures**

The in-depth interviews with policymakers and academic researchers regarding the implication of the policy measures indicated the following findings:

Lack of evidence regarding Monitoring policy: One of the marked issues was of a lack of continuous monitoring of the smoking situation in the country. This has given rise to policy formulation which is not backed by evidence generated through updated research on the scenario of smoking in the country.

"In our country, the research doesn't happen. We do not have the data whether the smokers have increased or decreased in this year in comparison to last year" (NGO Manager, Jhapa)

"We do not have the data on whether the number of smokers has increased or decreased in this year in comparison to last year. How much cigarette sales and consumption are happening? We don't need research to find out if cigarettes in harmful, everyone knows that. We need research to see if what we are doing to stop tobacco use is working or not" (Academic researcher, Kathmandu)

**Lack of relevant and applicable definition of open spaces in rural settings**

A ban on smoking in public places was intended to be a method of protecting nonsmokers from the harmful effects of smoking, while, decreasing the visibility of smoking to discourage initiation of smoking among the younger population. However, in the rural areas of Nepal, it was felt that this policy was not very relevant and applicable.

"Because our urban settings are very less, we have more rural setting. Focus is also more in rural areas. Rural areas are hill, mountain, and terai where there are many open spaces. "(Policy maker, Jhapa)

"People don't know how it works but they don't know how open field is to be controlled. They are confused. That is what is confusing about this law about cigarette smoking from my side" (Academic researcher, Kathmandu)

"How much is the fine and who takes the fine and where does it go? People ask this question when someone asks for a fine and the policy has no answer because there is no proper law, its only on paper" (Academic researcher, Kathmandu)

Inadequate Availability and Accessibility of cessation Facilities: The interviews indicated that the presence of smoking cessation facilities and options are inadequate for students, let alone in the schools. Any smokers willing to quit have poor, or no access to cessation services. A private quitline is available but information regarding this is not properly disseminated, so the people are not aware of its functionality, or the kind of support/counseling it provides.

"It would be good to have cessation corners like Counselling corners for drugs and alcohol in the schools, maybe provided at school levels by the government" (Policy maker, Jhapa)

"Any option at this point which would give smokers options to help them quit smoking would be helpful. If smoking initiation is rising and smokers are increasing, an option for quitting is mandatory as a form of help through policies" (Academic researcher, Kathmandu)

**Effectiveness of the Health Warning Labels (HWL)**

The findings indicated the lack of rotation of the health warning labels, lacking research on the effect of these labels, and whether the desired outcome was achieved through this policy.

"The first time, the picture on the packet is scary, but they see it every week now so they laugh at it now. Why isn't it being changed? Are there no more scary pictures?" (NGO manager, Jhapa)

"Oral cancer photo and lung cancer photo things no? That has been really given a good hate for the people about the use of tobacco. And I think this should be continued." (Policy maker, Kathmandu)

**Inadequate ban on advertising and sales of cigarettes to minors**

Age restrictions in the purchasing and selling of tobacco are lenient. An identification mandate is in place but not followed by the local shops and cigarette vendors, making cigarettes easily accessible to the younger population.

"In 100 meters radius, it is clearly stated that tobacco products are not to be sold or distributed. But you go, if you go anywhere near any temple, near any school or college, it will be sold nearby."(NGO Manager, Kathmandu)

"There are no strict rules and regulations. They can buy wherever without any ID and in loose sticks as well." (Academic researcher, Jhapa)

**Low taxation on cigarettes:** An increase in the cost of cigarettes is a very sustainable and cost-effective method for the government to raise revenue while targeting smokers directly and effectively. Nepal has one of the lowest taxation on tobacco products and cigarettes.

"I think, rather than others, taxation is not much implemented. The part of implementation is a bit loose in our country. If it were good, we can do it by increasing taxation."(Policy Maker, Kathmandu )

"Tax is the single most cost-effective way to fight tobacco for the country, yet there is a lot of unwillingness, which we do not understand" (Academic researcher, Jhapa)

**Quantitative results:** Among the total of 306 respondents, 148 (49.30%) were females and the rest were males. A total of 123 (40.80%) were in Grade 9, 99 (32.40%) in Grade 8, and 32.40 (26.80%) in Grade 10. It was reported that a total of 25 (8.10%) of respondents were smokers, of which 13 (4.20%) were current smokers and 12 (3.90%) were ever smokers. Of the total, 98 (32.10%) were of age 13 years, 106 (34.67%) were 14 years old and 102 (33.32%) were 15 years old with mean±standard deviation of 14.01 ±0.80 years and mean±standard deviation of age if initiation of smoking of 12.82 ±0.6 years. Out of 306 respondents, 81 (26.50%) of the respondents' parents smoked (Table 1).

**Table 1:** Participant characteristics of respondents (n= 306).

Characteristics	n (%)
**Age**	
13 years old	98 (32.10)
14 years	106 (34.67)
15 years	102 (33.32)
**Gender**	
Female	148 (49.30)
Male	155 (50.70)
**Grade**	
8	99 (32.40)
9	123 (40.80)
10	82 (26.80)
**Area of residence**	
Jhapa	150 (51)
Kathmandu	156 (49)
**Smoking status of parents**	
Yes	81 (26.50)
No	225 (73.50)
**Best friend smoking status**	
Definitely not	285 (93.10)
Probably not	17 (5.60)
Probably yes	2 (0.61)
Definitely yes	2 (0.70)

A total of 3.9% reported that they have tried smoking at least once in their lives (ever smokers). Among the total respondents (n=306), 25 (8.10%) were smokers among which, 13 (4.2%) were current smokers and 12 (3.9%) were ever smokers (Table 2).

**Table 2:** Smoking status of respondents (n= 306).

Smoking status of respondents	n (%)
Current smokers	13 (4.20)
Ever smokers	12 (3.90)
Never smoker	281 (91.90)

Among the current smokers, 53.8% of current smokers were in Grade 8, 23.04% in Grade 9 and Grade 10, and 46.1% of current smokers were of age 14 years. Among the total respondents, 1.6% reported that they would like to quit smoking sometime and even tried to quit smoking in the past 12 months and 66.7% reported that quitting smoking would be very difficult on a scale of 1-5 Likert scale.

In concurrence with the analysis of the qualitative interviews, despite the implementation of MPOWER policies since 2010, a total of 20.7% of respondents reported that someone smoked in their home, in front of them in the past 30 days. Of the respondents, 34.3% reported that they were exposed to second hand smoke in areas besides their homes, like schools, shops, restaurants, and other enclosed public spaces. A total of 50.3% also reported that they were audience to someone smoking within the school premises in the past 30 days. Also, 88.2% of the respondents mentioned that they think Second hand smoke is "Definitely harmful" to them if exposed. A total of 85% of students reported that they are in favor of banning smoking inside enclosed public places /outdoor places such as schools, shops, restaurants, shopping malls, and movie theaters. The interviews revealed that there is a lack of cessation services, which was in line with the findings of the survey, as reported by respondents in the quantitative survey. A total of 56.9% reported that they are aware of cessation clinics, Quitlines, NRT, and counselling being available in their locality and the remaining mentioned that they were not aware of any such service. A total of 3.9% of respondents reported that they purchased cigarettes in individual packs and 15.4% reported that they were not refused purchasing cigarettes because of their age at local shops and the remaining did not attempt to buy cigarettes.

The interviews revealed that the regulation banning the sale of individual sticks of cigarettes was not levied strictly in Nepal, along with the ban on selling cigarettes to anyone who is not of legal age. In concurrence, the quantitative survey revealed that none of the respondents reported that they were refused to purchase cigarettes because of their age. It was seen that the mean age of initiation of smoking was 12.82 years (0.60) Also, the mean expenditure on cigarettes monthly was NRs. 10.807 among the 306 respondents.

## DISCUSSION

For the first time, global smoking prevalence has decreased.^10^ Still, studies show that there are 1.3 billion global tobacco users worldwide and it remains a leading cause of death and diseases. It was reported that a total of 8.1% of respondents were smokers, from among which 4.2% were current smokers and 3.9% were ever smokers. This is lower than the previously reported prevalence of any tobacco use among adolescents (37%) in the eastern region of Nepal.^3^ Since this study has a lower sample size, the findings may have been reportedly lower than the previous national survey. However, multiple studies have been conducted in the past to assess the smoking status among individuals aged 15-49 years through the GATS, but there is a consistent lack of data reporting the smoking status of adolescents below 15 years of age.^2^ A possible reason can be the lack of regular GYTS in the country over time. Through this study, it was reported that the proportion of male smokers was higher than that of females. This can be owed to possible underreporting of this gender as seen in the past through literature in most parts of the world that females smoke less, but also report less even if they smoke due to certain cultural and societal issues that they face in their countries.^3^

**Qualitative:** The interviews revealed that urban areas like Kathmandu show higher patterns of smokers although the policies have been disseminated rather well when compared to the rural areas of the country. Factors like in-country migration from all over the country into Kathmandu, however, pose a doubt in the actual representation of the smokers being actually from the city. Interviews with stakeholders from our study found that the enforcement of tobacco control laws is more strict in cities like Kathmandu and more lax in rural areas like Jhapa. There has also been speculation about the implementation of smoking bans in rural areas, where there may not be a proper demarcation of public and private areas. There was less compliance in rural areas as reported by literature as well.^8^ In A study from Pokhara, Nepal, showed that most of the respondents 66.7% smoked in public places like tea stalls or restaurants.^9^ This still exists and it can be due to the fact that smoking in public places has been banned in the country since the past decade but is not implemented properly.

**Quantitative:** The mean age for smoking initiation (smoking and chewing) in this study was found to be consistent with studies from Kathmandu, Noida, and Kerala, India where the mean ages of onset were^14,15^ 12.4 and 13.2 years, respectively.^10^ It has been reported that the required attention is not being emphasized for this MPOWER measure. In our study, a total of 79.7% of respondents reported that they were exposed to some kind of anti-tobacco media messages on television, radio, internet, billboards, posters, newspapers, magazines, or movies in the past 30 days. Among the respondents, 66% mentioned that they had seen health warning labels in cigarette packets in the past 30 days. On qualitative assessment, it was reported that, on a more contextual front, perhaps in adjunct to HWL's, role play, street drama, and theatre would be more effective, especially in the rural side of the country. This can be, as also reported that on comparison of the Nepalese warning label with other foreign labels with regards to providing knowledge of harm warning, the impact of quitting smoking, and giving cigarettes as a gift, the overseas labels were found to be more effective.^11^

Since the introduction of the WHO-FCTC more than a decade ago, countries like Nepal, have improved their taxation standards, but not high enough for youths. Tobacco taxation has been an essential component of a comprehensive tobacco control strategy.^12^ Most studies found that raising cigarette prices through increased taxes is a highly effective measure for reducing smoking among youth, young adults, and persons of low socioeconomic status. More disturbingly, cigarettes are about 60% more affordable than they were at the beginning of the 1970s.^12^

This study selected students based on the study's inclusion and exclusion criteria and sampling strategy which could have led to selection bias, so the study may not be generalizable to the entire student's population of the country.

At policy level, adequate enforcement of policy on increasing the price of cigarettes through the WHO benchmark of 70% taxation is recommended to address the affordability and accessibility of cigarettes is recommended. The demarcation of rural and urban areas should be clear for the people to understand and follow. Similarly, fines and collection along with designated authority for this act is recommended for better enforcement of the Protect people from SHS policy. A strict ban on purchase of cigarettes to students <18 years of age should be practiced with fine on shops who do not comply by this rule. To address the smoking in this age group, cessation corners and counselling should be available in schools to provide help whenever any student wants to avail this service.

In order to provide evidence for policy level decision making, periodic GYTS and policy analyses is required in order to monitor and assess the outcome of tobacco control policies in Nepal to evaluate the successes and failures of the policies. Future studies to explore various other types of tobacco uses is recommended.

Further, different research methods such as sequential exploratory mixed methods, ethnography, properly designed randomized controlled trials to test the effectiveness of any interventions addressing smoking prevalence and incidence.

## CONCLUSIONS

A decade after the WHO-FCTC policy came into effect in Nepal, implementation gaps are quite visible. Protobacco advertisements are still visible, cigarettes are still affordable, a ban on public smoking is not practiced efficiently and fines are not levied at all. Nepal is still one of the countries with the lowest tax on cigarettes and tobacco products. Policies that may have proven effective in a high-income country may or may not give the same results in a low- or middle-income country. The policies are the same, but the modality and prioritization of their implementation should be contextual to the country
